# Cross-sectional evaluation of cardiovascular biological age using point-of-care ultrasound

**DOI:** 10.1093/ehjdh/ztag047

**Published:** 2026-03-19

**Authors:** Roi Amster, Abigail Goshen, Harel Raanani, Adiel Am-Shalom, Michael Fiman, Robert Klempfner, Ehud Raanani, Ehud Schwammenthal, Evelyne Bischof, Elad Maor, Tzipora Strauss

**Affiliations:** Sheba Longevity Center, Sheba Medical Center, Ramat Gan, Israel; The Leviev Cardiothoracic and Vascular Center, Sheba Medical Center, Ramat Gan, Israel; Faculty of Medicine, Tel-Aviv University, Tel Aviv, Israel; Sheba Longevity Center, Sheba Medical Center, Ramat Gan, Israel; Department of Management, Health Systems Management Program, Bar-Ilan University, Ramat Gan, Israel; Faculty of Medicine, Tel-Aviv University, Tel Aviv, Israel; Aisap.ai, Israel; Aisap.ai, Israel; Aisap.ai, Israel; The Leviev Cardiothoracic and Vascular Center, Sheba Medical Center, Ramat Gan, Israel; Faculty of Medicine, Tel-Aviv University, Tel Aviv, Israel; Aisap.ai, Israel; The Leviev Cardiothoracic and Vascular Center, Sheba Medical Center, Ramat Gan, Israel; Faculty of Medicine, Tel-Aviv University, Tel Aviv, Israel; Aisap.ai, Israel; The Leviev Cardiothoracic and Vascular Center, Sheba Medical Center, Ramat Gan, Israel; Faculty of Medicine, Tel-Aviv University, Tel Aviv, Israel; Aisap.ai, Israel; Sheba Longevity Center, Sheba Medical Center, Ramat Gan, Israel; Faculty of Medicine, Tel-Aviv University, Tel Aviv, Israel; The Leviev Cardiothoracic and Vascular Center, Sheba Medical Center, Ramat Gan, Israel; Faculty of Medicine, Tel-Aviv University, Tel Aviv, Israel; Sheba Longevity Center, Sheba Medical Center, Ramat Gan, Israel; Faculty of Medicine, Tel-Aviv University, Tel Aviv, Israel

**Keywords:** Cardiovascular disease, Ultrasound-based cardiovascular ageing clock, Biological age, Point-of-care ultrasound

## Abstract

**Aims:**

Biological age is increasingly recognized as a superior predictor of morbidity, mortality, compared with chronological age. Artificial intelligence (AI)-driven ageing clocks enable rapid, non-invasive assessment. Cardiovascular (CV) ageing is of particular relevance given its central role in systemic metabolic health. This study evaluated the clinical utility of an ultrasound (US)-based CV biological age clock derived from handheld point-of-care ultrasound (POCUS), in comparison with haematological and electrocardiographic (ECG)-based clocks.

**Methods and results:**

We analysed 243 adults (median age 62 years; 54% women) from the Sheba Healthspan Research Population (SHARP) study. Ultrasound-based CV age was estimated using focused cardiac POCUS with AI software. Blood age was calculated using the SenoClock platform from 45 routine biomarkers, and ECG age was derived using a convolutional neural network trained on >770 000 tracings. Correlations with chronological age and inter-clock agreement were examined. Participants were stratified into quintiles of US delta (US–chronological age). All three clocks correlated with chronological age (blood: *r* = 0.89, US: *r* = 0.74, ECG: *r* = 0.61; all *P* < 0.001). US-accelerated agers (top quintile) displayed a more adverse cardiometabolic profile, including higher diastolic blood pressure, body mass index, waist circumference, triglycerides, alongside lower HDL cholesterol, and more than double the prevalence of metabolic syndrome. Those with US age ≥2 years above chronological age had significantly higher odds of metabolic syndrome (odds ratio = 2.34, 95% confidence interval: 1.07–5.17, *P* = 0.034).

**Conclusion:**

AI-derived ultrasound-based cardiovascular biological age from handheld POCUS is associated with prevalent metabolic syndrome in this cross-sectional cohort, even when routine focused POCUS shows no abnormalities warranting referral.

## Graphical Abstract

**Graphical Abstract:**
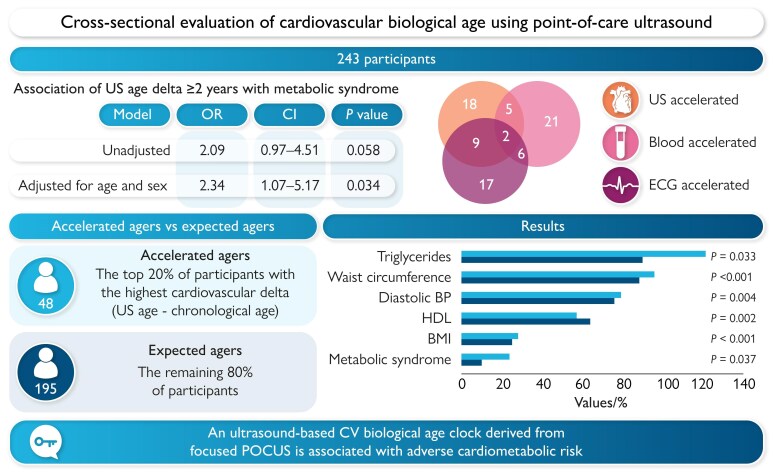

## Introduction

Biological age, distinct from chronological age, is increasingly recognized as a more accurate indicator of morbidity, mortality, and physiological resilience. It captures interindividual variation in ageing trajectories shaped by genetic, behavioural, and environmental factors, and often surpasses chronological age in predicting health outcomes.^1–3^ Diverse biological age clocks—based on epigenetic, haematological, metabolic, and imaging modalities—have demonstrated varying utility in forecasting disease risk and lifespan.^4–7^ Among these, cardiovascular ageing clocks have emerged as particularly compelling, given the central role of cardiovascular integrity in ageing biology and its tight linkage to systemic metabolic health.^8,9^

Existing modalities for cardiovascular age estimation, however, face several limitations. Conventional echocardiography, though highly informative, is time-intensive, operator-dependent, and not easily scalable. In contrast, point-of-care ultrasound (POCUS) has transformed bedside evaluation by offering a rapid, non-invasive window into cardiovascular function, particularly in acute care settings.^10–13^ Yet POCUS alone suffers from interpretative variability and requires considerable training, limiting its application for standardized biological ageing assessment.^10,14^

Recent advances in artificial intelligence (AI) have begun to bridge this gap. AI-enhanced interpretation of cardiac ultrasound images has shown high diagnostic accuracy for detecting subclinical cardiac dysfunction and stratifying cardiovascular risk in a reproducible, operator-independent manner.^15–19^ Building on these foundations, cardiovascular biological age derived from AI-powered POCUS now offers an accessible, scalable, and physiologically relevant biomarker. Critically, recent work suggests that a higher cardiovascular biological age particularly when discordant from chronological age predicts adverse clinical outcomes, including excess cardiovascular mortality.^20^

This study introduces and evaluates an AI-based, handheld ultrasound-derived cardiovascular age clock. We compare this modality against two other validated biological ageing tools: A haematological ageing clock (blood-based), and a neural network-based electrocardiographic (ECG) ageing clock. By leveraging comprehensive clinical and metabolic profiling, we examine the interrelationships, divergences, and clinical correlates of each clock’s outputs.

Our central aim is to assess whether AI-augmented cardiovascular biological age captured at the bedside in under 5 min can serve not only as a feasible and scalable tool but also as a distinct and clinically meaningful indicator of subclinical cardiometabolic stress. We explore the degree to which this clock is associated with high-risk phenotypes, its correlation with traditional risk markers, and its potential role in risk stratification strategies. This work contributes to the growing body of literature advocating for biologically anchored, AI-driven tools to guide early detection and intervention in age-related disease

## Methods

### Study population and design

The Sheba Healthspan Research Population (SHARP) study is a prospective, 12-month randomized controlled trial conducted at the Sheba Longevity Center, aimed at evaluating the impact of personalized health and behavioural interventions on biomarkers of ageing. Self-referred, healthy adults aged >50 years undergo comprehensive baseline assessments and follow-up visits, including extensive clinical, physical, cognitive, and functional evaluations. All participants were recruited via the official website of Sheba Longevity Center and provided written informed consent. A subset of SHARP participants was included in the current cross-sectional sub-study, evaluating three biological ageing clocks at baseline: (i) ultrasound-based cardiovascular age derived from focused cardiac point-of-care echocardiography, (ii) haematological (‘blood’) age calculated from blood biomarkers, and (iii) AI-derived electrocardiographic (ECG) age.

### Data collected

#### Echocardiographic assessment of cardiovascular biological age

Participants underwent a focused, point-of-care ultrasound (POCUS) cardiac assessment using a handheld Philips Lumify ultrasound device integrated with an AI-driven software developed by Aisap.ai (Israel), in collaboration with Sheba Medical Center. The model brings an ultrasound diagnostic assessment software, and combines AI diagnostics and detailed measurements for rapid and accurate diagnosis of structural heart disease and heart failure at the bedside by non-cardiologists, as previously described.^19,20^ Participants were scanned in the left lateral decubitus position, capturing only two standard echocardiographic views: parasternal long-axis and apical four-chamber. Image acquisition typically lasted under five minutes. The AI model provided an estimation of cardiovascular biological age based on echocardiographic features. The AI model analyses raw B-mode echocardiographic video loops (parasternal long-axis and apical four-chamber) rather than pre-extracted volumes or operator-dependent measurements. The model architecture combines the use of convolutional neural networks (CNN) and transformers to achieve the required output. The model was trained and validated on more than 98 000 transthoracic echocardiograms annotated for chronological age and tested on more than 22 000 exams (as reported in Faierstein *et al*., JASE 2024,^20^) achieving a mean absolute error of 4.9 years, with a Pearson correlation coefficient of 0.922 in an independent validation cohort. In this study of 258 participants initially scanned, 243 (94%) had echocardiographic images suitable for AI analysis and comprised the final cohort.

#### Blood biological age prediction

Haematological (blood) biological age was calculated using the SenoClock-BloodAge, an established haematological ageing clock developed by Deep Longevity (Hong Kong, subsidiary of Regent Pacific 00 575.HK).^21^ SenoClock is an ensemble of 21 deep neural networks trained on data from over 60 000 individuals. The model utilized 45 routinely measured blood biomarkers, including albumin, haemoglobin, haematocrit, RBC indices, WBC subtypes, platelets, electrolytes (sodium, potassium, chloride, calcium, phosphorus), renal function markers (blood urea nitrogen, creatinine), liver function tests (total protein, globulin, bilirubin, alanine transaminase, aspartate transaminase, gamma-GT, alkaline phosphatase), and lipid parameters. The analysis was performed via the online SenoClock platform (https://www.deeplongevity.com/senoclock).

#### AI-ECG biological age prediction

ECG-derived biological age was determined using a CNN developed by Attia *et al*.^22^ The CNN, constructed in Keras with a TensorFlow backend (Google, Mountain View, CA, USA), was trained on ECG data from 774 783 unique subjects from the Mayo Clinic digital archive.^23^ The architecture consisted of convolutional, max-pooling, batch normalization layers, and fully connected layers. Model input included standard 10-s, resting 12-lead digital ECG tracings, producing an age prediction as a continuous variable (years).^24^

#### Additional covariates

Additional covariates included socio-demographic characteristics (sex, years of education, chronological age), lifestyle factors (smoking status, physical activity), body composition measures [body mass index (BMI), waist circumference, fat percentage], blood pressure (systolic, diastolic), lipid profile [total cholesterol, low-density lipoprotein (LDL), high-density lipoprotein (HDL), triglycerides, apolipoprotein B], inflammatory marker [C-reactive protein (CRP)], and clinical diagnoses [cardiovascular disease (CVD), metabolic syndrome]. Metabolic syndrome was defined according to commonly accepted criteria,^25^ requiring the presence of at least three of the following five components: triglycerides ≥150 mg/dL; fasting plasma glucose ≥100 mg/dL; reduced HDL cholesterol (<40 mg/dL in men and <50 mg/dL in women); increased waist circumference (>102 cm in men and >88 cm in women); and elevated blood pressure (systolic ≥130 mmHg and/or diastolic ≥85 mmHg).

#### Statistical analysis

Continuous variables are presented as means ± standard deviations (SD), and categorical variables as frequencies and percentages. Between-group comparisons were conducted using independent-samples *t*-tests for continuous variables and chi-square tests for categorical variables. All tests were two-sided, and statistical significance was defined as *P* < 0.05. Correlations between chronological age and biological clocks (US, blood, ECG), as well as inter-clock correlations, were assessed using Pearson’s correlation coefficients for normally distributed data and Spearman’s rank correlation otherwise. Results are presented with correlation coefficients (*r*) and corresponding *P*-values.

Given established age-dependent bias in biological age prediction models, all analyses involving age-delta (ΔAge) were performed using bias-corrected estimates to ensure independence from chronological age. Age-dependent bias was formally evaluated prior to downstream analyses, and correction was applied before group stratification, correlation analyses, and regression modelling. Two established correction approaches were implemented: Cole’s method, which linearly rescales predicted age to enforce unit slope with the identity line, and the offset-based method proposed by Beheshti *et al*., which models age-delta as a function of chronological age and removes the estimated age-dependent component.^26,27^ Corrected biological age and corresponding ΔAge values were derived for each method, and residual age dependency was evaluated using linear regression diagnostics. All analyses involving ΔAge were conducted using the Beheshti bias-corrected estimates.

To evaluate accelerated vs. expected ageing, participants were stratified into quintiles based on bias-corrected ΔAge for each clock. Quintile 1 represented ‘super agers’ (biologically younger), whereas Quintile 5 represented ‘accelerated agers’ (biologically older), terms previously used in the context of longevity by Topol.^28^ Characteristics across quintiles were compared using one-way ANOVA for continuous variables and Chi-square tests for categorical variables. Particular focus was placed on Quintile 5 (‘accelerated agers’) compared with all others (‘expected agers’).

Cross-clock concordance was evaluated using cross-tabulations and chi-square tests. Cohen’s κ was calculated to quantify agreement beyond chance. Venn diagrams were used to illustrate overlap between accelerated agers defined by different clocks.

Finally, to assess the potential of the US-based CV biological age clock clinical relevance, additional logistic regression analyses were conducted. Specifically, we examined whether exceeding predefined thresholds of US ΔAge was associated with metabolic syndrome using bias-corrected ΔAge estimates. Models included an unadjusted analysis, adjustment for chronological age, and full adjustment for chronological age and sex. This approach enabled estimation of odds ratios (ORs) and 95% confidence intervals (CIs) for clinically meaningful cut-offs (e.g. ≥2 years above chronological age), thereby complementing the quintile-based framework. In addition, as a sensitivity analysis, bias-corrected US ΔAge for each biological clock (US, blood, and ECG) was also evaluated as a continuous predictor (per 1 SD) in logistic regression models to allow a modality-specific comparison across clocks.

Analyses were performed on a complete-case basis; participants with missing data for a given clock or variable were excluded from analyses specific to that variable. No data imputation was conducted. Statistical significance was defined as two-sided *P* < 0.05. Analyses were conducted using SPSS (version 26.0, IBM Corp.) and R (version 4.3.1).

## Results

The final cohort comprised 243 participants (median age: 62 years, IQR: 56–68), with 131 (54%) women. Participants were stratified into quintiles according to the bias-corrected delta between US-based CV biological age and chronological age, where Quintile 1 represented biologically ‘super agers’ and Quintile 5 represented ‘accelerated agers’. Details of the age-bias correction procedures are provided in Appendix Supplementary material online, *Figure S1*. The full distribution of clinical and demographic characteristics across quintiles is provided in Supplementary material online, Appendix  *Table S1*. On focused POCUS examination, most participants showed no echocardiographic findings warranting clinical referral. In quintile 5, 45/49 (91.8%) had no abnormal findings compared with 183/194 (94.3%) in quintiles 1–4, with no significant difference between groups (χ^2^(1) = 0.42, *P* = 0.517).

***Table 1*** compares baseline characteristics of accelerated agers (Q5, *n* = 48) with all remaining participants (Q1–Q4, *n* = 195). Following bias correction, the two groups did not differ significantly in chronological age (60.9 ± 6.6 vs. 62.4 ± 8.7 years, *P* = 0.20). Despite similar chronological age, accelerated agers demonstrated a significantly less favourable cardiometabolic profile. They had higher diastolic blood pressure (79.9 ± 8.3 vs. 75.8 ± 8.4 mmHg, *P* = 0.004), BMI (27.9 ± 3.6 vs. 25.1 ± 3.8 kg/m^2^, *P* < 0.001), waist circumference (97.6 ± 9.3 vs. 89.5 ± 11.4 cm, *P* < 0.001), triglycerides (117.3 ± 78.7 vs. 91.3 ± 49.0 mg/dL, *P* = 0.03), and more than double the prevalence of metabolic syndrome (23% vs. 10%, *P* = 0.03). Conversely, they had lower HDL cholesterol (57.0 ± 13.1 vs. 64.0 ± 15.6 mg/dL, *P* = 0.002). No significant differences were observed in systolic blood pressure, total cholesterol, LDL cholesterol, apolipoprotein B levels, C-reactive protein, smoking status, physical activity, body fat percentage, or prior CVD history.

**Table 1:** Baseline characteristics: ‘accelerated agers’ (fifth quintile) vs. other quintiles according to age-bias–corrected US-based cardiovascular age difference (ΔAge)

Characteristic	Overall	Expected agers (Q1–Q4)	Accelerated agers (Q5)	*P*-value
	*N* = 243	*N* = 195	*N* = 48	
**US age (mean, SD)**	62.11 (10.07)	60.56 (10.19)	68.40 (6.56)	<0.001
**Mean difference (SD)**	0.00 (5.70)	−1.85 (4.69)	7.53 (2.13)	<0.001
**Age (mean, SD)**	62.11 (8.30)	62.41 (8.66)	60.87 (6.59)	0.2
**Sex**				0.039
** Male**	112 (46%)	83 (43%)	29 (60%)	
** Female**	131 (54%)	112 (57%)	19 (40%)	
**Education (years)**	15.62 (3.38)	15.68 (3.34)	15.39 (3.56)	0.7
**Smoking**				>0.9
** Never**	92 (39%)	73 (38%)	19 (40%)	
** Smoker (current/past)**	146 (61%)	118 (62%)	28 (60%)	
**Systolic BP**	125.57 (16.86)	124.98 (17.17)	127.98 (15.47)	0.3
**Diastolic BP**	76.57 (8.48)	75.77 (8.36)	79.87 (8.27)	0.004
**Total cholesterol**	185.64 (42.28)	185.80 (42.99)	184.98 (39.66)	>0.9
**LDL cholesterol**	105.59 (35.46)	105.17 (35.38)	107.29 (36.11)	0.7
**HDL cholesterol**	62.58 (15.35)	63.97 (15.59)	56.96 (13.06)	0.002
**Triglycerides (TG)**	96.48 (56.94)	91.32 (49.05)	117.29 (78.70)	0.033
**Apo B**	93.56 (25.49)	92.62 (25.15)	97.38 (26.76)	0.3
**CRP**	2.23 (4.12)	1.89 (2.43)	3.60 (7.77)	0.14
**BMI**	25.68 (3.90)	25.14 (3.78)	27.93 (3.60)	<0.001
**Waist circumference**	91.08 (11.48)	89.46 (11.41)	97.64 (9.27)	<0.001
**Fat %**	32.86 (8.08)	33.04 (8.08)	32.08 (8.13)	0.5
**Previously diagnosed CVD^a^**	53 (22%)	40 (21%)	13 (27%)	0.568
**Metabolic syndrome**	31 (13%)	20 (10%)	11 (23%)	0.037

^a^Includes hypertension, diabetes, ischaemic heart disease (IHD), or dyslipidaemia

There was no statistical difference in the prevalence of reduced ejection fraction (EF < 55%) between accelerated agers (8.3%) and all other participants (9.6%). No statistical differences were observed between the groups in other echocardiographic features measured; left atrial area, right atrial area, pericardial effusion, right ventricular function, aortic root diameter, left ventricular end diastolic diameter, right ventricular fractional area change.

These findings indicate that membership in the top quintile of US-based CV biological ageing captures individuals who are metabolically older, highlighting the clock’s association with prevalent cardiometabolic disease.

### Correlations between biological and chronological ages

All three biological age clocks demonstrated strong associations with chronological age when evaluated using the raw predicted age estimates (***Table 2*)**. The blood clock showed the strongest correlation (*r* = 0.89, *P* < 0.001), followed by the US clock (*r* = 0.74, *P* < 0.001), and the AI-ECG clock (*r* = 0.61, *P* < 0.001). The clocks were also significantly intercorrelated: US vs. blood (*r* = 0.66), US vs. ECG (*r* = 0.62), and blood vs. ECG (*r* = 0.59; all *P* < 0.001). These results underscore that while each clock tracks chronological age, the degree of correlation varies, suggesting partially distinct biological information captured by each modality.

**Table 2:** Correlation matrix among different ageing clock calculations^a^

	Chronological age	US age	Blood age	AI-ECG age
**Chronological age**	1	0.735	0.885	0.614
**US age**	0.735	1	0.660	0.616
**Blood age**	0.885	0.660	1	0.586
**AI-ECG age**	0.614	0.616	0.586	1

^a^All Correlation presented are significant at the 0.001 level (two-tailed).

### Cross-clock concordance

**Figure 1:**
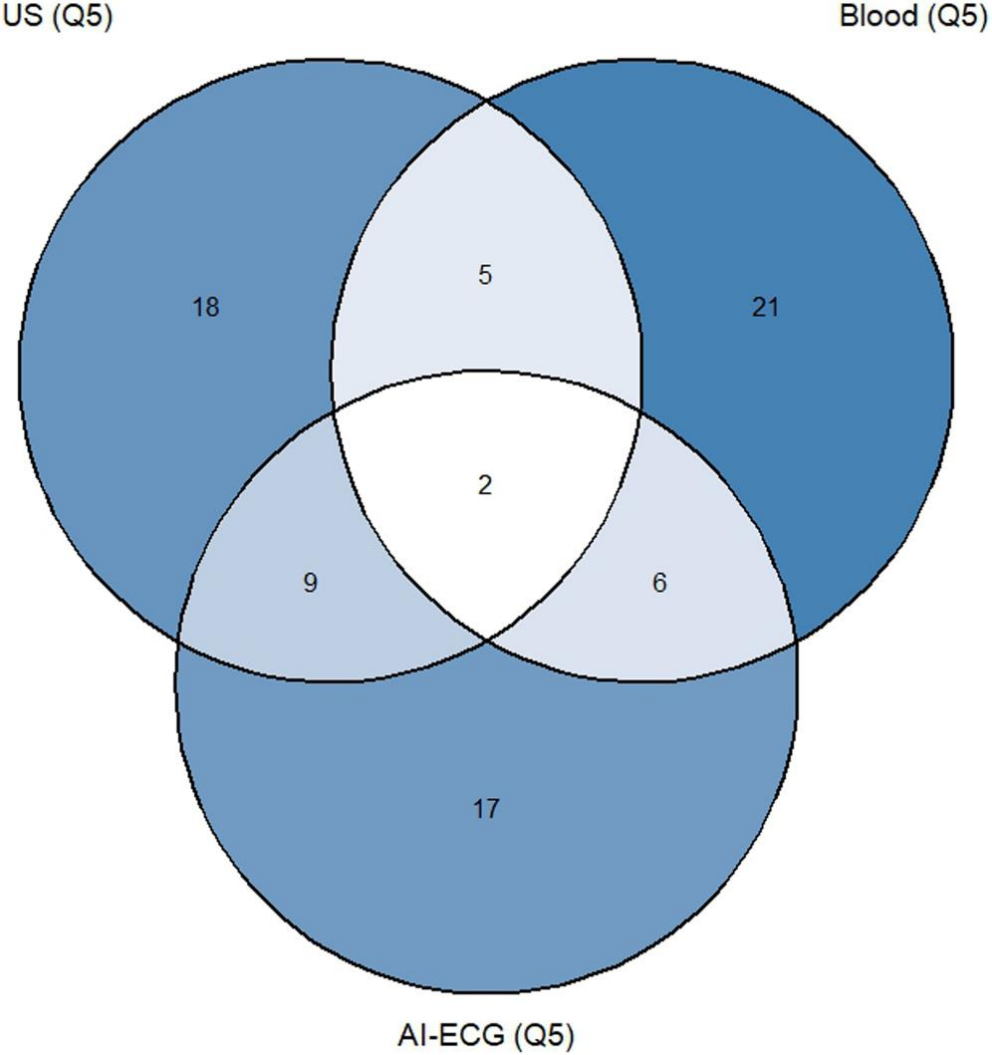
Venn diagram presenting cross-clock overlap of accelerated agers after age-bias correction. Venn diagram illustrating the overlap in classification of accelerated agers (fifth quintile, Q5) across three biological age clocks: US-based cardiovascular age, blood-based age, and AI-ECG age. Classification was based on quintiles of bias-corrected ΔAge (Beheshti method). Numbers within each region indicate the count of participants classified as accelerated agers by each clock alone or jointly. Only a small subset of participants was consistently identified as accelerated agers across all three clocks, whereas the majority were uniquely classified by a single clock, highlighting limited concordance and the distinct biological domains captured by each ageing modality. Complete-case analysis across all three clocks included 176 participants.

***Table 3* and *Figure 1*** present cross-tabulations and Venn diagrams assessing concordance in the classification of accelerated agers using age-bias–corrected ΔAge measures across clocks.

**Table 3:** Cross-clock concordance of accelerated and expected agers based on age-bias–corrected biological age estimates

	Blood age Expected agers *N* (%)	Blood age accelerated agers *N* (%)
**US age expected agers**	158 (66.7%)	32 (13.9%)
**US age accelerated agers**	32 (13.5%)	15 (6.3%)

	AI-ECG age expected agers *N* (%)	AI-ECG age accelerated agers *N* (%)
**US age expected agers**	118 (66.7%)	24 (13.55%)
**US age accelerated agers**	24 (13.55%)	11 (6.2%)

	AI-ECG age expected agers *N* (%)	AI-ECG age accelerated agers *N* (%)
**Blood age expected agers**	114 (64.8%)	27 (15.3%)
**Blood age accelerated agers**	27 (15.3%)	8 (4.6%)

Among the 237 participants with both US and blood data, only 15 (6.3%) identified as accelerated agers by both clocks, while 64 (27%) were discordantly classified. This corresponded to a statistically significant association (χ^2^ = 5.38, df = 1, *P* = 0.020), although the concordance was minimal with Cohen’s Kappa (κ = 0.15), indicating slight agreement. Similarly, concordance between US and ECG clocks (n = 177) was modest, with 11 participants (6.2%) concurrently classified as accelerated agers, whereas 48 (27.1%) were discordant. The association did not reach statistical significance (χ^2^ = 3.74, df = 1, *P* = 0.053), again with only slight agreement (κ = 0.15). By contrast, overlap between blood and ECG clocks was minimal (*n* = 176), with only 8 participants (4.6%) classified by both while 54 (30.7%) were discordantly classified. No significant association was observed (χ^2^ = 0.24, df = 1, *P* = 0.623), and agreement was negligible (κ = 0.04). Overall, the Venn diagram (*Figure 1*) illustrates that the majority of accelerated agers were uniquely identified by a single clock, emphasizing the distinct physiological domains captured.

### Metabolic syndrome and accelerated agers across clocks

Participants in the top quintile of the US-based cardiovascular (CV) bias-corrected ΔAge demonstrated substantially higher prevalence of metabolic syndrome. When applying a clinical cut-off of ≥2 years, individuals above this threshold exhibited significantly elevated odds of metabolic syndrome, corresponding approximately to one standard deviation of the US ΔAge distribution, rather than the full range of acceleration observed within the upper quintile.

Using this predefined threshold, individuals with US ΔAge ≥2 years exhibited significantly elevated odds of metabolic syndrome (***Table 4***). In the unadjusted analyses, the association didn't reach statistical significance (OR = 2.09, 95% CI: 0.97–4.51, *P* = 0.058). After adjustment for chronological age and sex, the association strengthened and reached statistical significance (OR = 2.34, 95% CI: 1.07–5.17, *P* = 0.034). In contrast, no significant associations were observed for the Blood-based or AI-ECG clocks when applying the same categorical threshold (ΔAge ≥2 years), either in unadjusted models or after adjustment for chronological age and sex. As an additional sensitivity analysis, ΔAge for each biological clock (US, blood, and ECG was also evaluated as a continuous predictor (per 1 SD increase, **Appendix**  Supplementary material online, ***Table S2***) of metabolic syndrome. Logistic regression models; unadjusted, age-adjusted, and fully adjusted for age and sex, showed that, higher continuous ΔAge was significantly associated with metabolic syndrome for the US clock (OR = 2.20, 95% CI: 1.41–3.58, *P* < 0.001) and more modestly for the blood clock (OR = 1.53, 95% CI: 1.06–2.21, *P* = 0.022), whereas no significant association was observed for the AI-ECG clock. These findings were consistent with the categorical analyses and further support the stronger cardiometabolic relevance of the US-based biological ageing measure.

**Table 4:** Association between bias-corrected ΔAge ≥2 years and metabolic syndrome^a^

Clock	Model	*N*	OR	95% CI	*P*
**US**	Unadjusted	241	2.09	0.97–4.51	0.058
**US**	Adjusted for age and sex	241	2.34	1.07–5.17	0.034
**Blood**	Unadjusted	236	1.3	0.54–2.95	0.539
**Blood**	Adjusted for age and sex	236	1.37	0.55–3.20	0.473
**AI-ECG**	Unadjusted	177	1.24	0.50–3.00	0.636
**AI-ECG**	Adjusted for age and sex	176	1.35	0.54–3.30	0.518

^a^Odds ratios (OR) and 95% confidence intervals (CI) are shown for unadjusted, and fully adjusted (age and sex) logistic regression models.

Taken together, these results indicate that a ≥ 2-year elevation in bias-corrected US-based CV biological age may serve a clinically meaningful threshold for identifying individuals at increased cardiometabolic risk, reinforcing the discriminatory value of the US-based CV clock beyond traditional chronological age.

## Discussion

This study provides novel evidence for the potential clinical utility of an ultrasound-based CV biological age clock derived from focused cardiac point-of-care ultrasound (POCUS). US-based CV clock is associated with adverse cardiometabolic profiles, whereas blood-based and ECG-derived clocks showed weaker or inconsistent relationships. Most participants had no echocardiographic findings that would warrant clinical referral, yet substantial variability in US biological age was detected, suggesting its ability to capture physiological differences not reflected in routine focused POCUS interpretation.

Accelerated agers defined by the US clock exhibited consistently worse cardiometabolic traits, including higher BMI, greater waist circumference, elevated diastolic blood pressure, higher triglycerides, higher prevalence of metabolic syndrome and lower HDL cholesterol. These associations were observed despite similar chronological age distributions between accelerated and non-accelerated groups, indicating that the US-based CV clock captures biological heterogeneity relevant to cardiometabolic health beyond age alone. A single US-based CV biological age identify patients with known cardiometabolic risk factors, such as metabolic syndrome, hypertension and dyslipidaemia. These results are consistent with prior work demonstrating increased long-term mortality among US-based CV accelerated agers using the same tool.^20^

Beyond quintile stratification, we identified a practical clinical threshold: participants whose US biological age exceeded their chronological age by ≥2 years had significantly higher odds of metabolic syndrome (OR = 2.34), even after adjustment for chronological age and sex. This cut-off provides an interpretable reference within the current cohort, although external validation is required before broader application. These findings suggest that US-based biological age reflects meaningful variation in cardiometabolic status rather than a purely statistical construct.

Although all three clocks correlated strongly with chronological age, concordance in identifying accelerated agers was weak (Cohen’s κ <0.20). This dissociation highlights that each clock captures distinct aspects of biological ageing. The blood clock reflects systemic and lipid-related processes, while the US clock appears to integrate structural and functional cardiac adaptations linked to metabolic stress. ECG-derived age demonstrated moderate associations. Thus, the clocks are complementary rather than redundant, and their combined use may enhance individualized risk assessment.

A key strength of the US clock is its dual role in a single brief examination. Every POCUS scan already provides standard echocardiographic information ventricular size and function, valvular integrity, ejection fraction, and stroke volume that guides routine clinical care. The same images, analysed by AI, simultaneously yield an estimate of US biological age. This additional layer of information requires no extra acquisition or laboratory testing and may provide complementary characterization of cardiometabolic phenotype within the context of a cross-sectional assessment, particularly in settings where comprehensive metabolic evaluation is not routinely available.

Several limitations must be acknowledged. First, as the study is cross-sectional, age-delta reflects associations with prevalent cardiometabolic traits rather than prediction of incident outcomes. Accordingly, the findings do not establish impact on clinical outcomes or preventive benefit, and longitudinal follow-up will be required to determine prognostic value and incremental utility beyond standard clinical evaluation. Second, the population consisted of health-conscious, self-referred volunteers, limiting generalizability. Broader, more diverse cohorts are needed to validate findings. Third, while feasibility was high overall (94%), image quality excluded a minority of scans. Fourth, reproducibility (test–retest reliability) was not assessed because repeat scanning was not performed; this should be evaluated in future work. Finally, although a clinically relevant ΔAge threshold was identified, external validation in independent cohorts is essential before considering broader clinical implementation.

### Conclusion and clinical implications

An AI-enhanced US-based CV biological age clock derived from focused POCUS is associated with prevalent cardiometabolic risk (metabolic syndrome) in this cohort, even when routine focused POCUS shows no abnormalities warranting referral. A difference of more than two years above chronological age was associated with substantially increased odds of metabolic syndrome. Crucially, this metric is generated opportunistically during a scan that already delivers standard echocardiographic parameters, offering additional information without extra testing; however, its clinical role requires prospective and external validation before routine implementation. Future work should determine its prognostic relevance in longitudinal cohorts, responsiveness to interventions, and incremental value beyond established risk scores.

## Supplementary Material

ztag047_Supplementary_Data

## Data Availability

Participant-level data are not publicly available due to institutional privacy and ethical restrictions. De-identified derived data supporting the findings of this study (including bias-corrected ΔAge values) are available upon reasonable academic request. The proprietary Aisap.ai model weights are not available due to regulatory and licencing constraints.
