# Supplementary material for: Cross-sectional evaluation of cardiovascular biological age using point-of-care ultrasound
Source: Eur Heart J Digit Health. 2026 Mar 19;7(3):ztag047. doi: 10.1093/ehjdh/ztag047 (PMC13026412; doi:10.1093/ehjdh/ztag047)
Supplement: ztag047_Supplementary_Data [file ztag047_supplementary_data.docx]

**Appendix Table S1. Baseline Characteristics Across Quintiles of Chorological Age –** **Beheshti Bias-Corrected US Age**

| Characteristic | Overall | Quintile 1 | Quintile 2 | Quintile 3 | Quintile 4 | Quintile 5 | p-value |
| --- | --- | --- | --- | --- | --- | --- | --- |
|  | N = 243 | N = 49 | N = 49 | N = 49 | N = 48 | N = 48 |  |
| Mean corrected Difference (SD) | 0.00 (5.70) | -8.33 (2.99) | -2.76 (0.96) | 0.31 (0.81) | 3.48 (1.21) | 7.53 (2.13) | <0.001 |
|  |  | **Super Agers** |  | **Normal Agers** |  | **Accelerated agers** |  |
| Age (mean, SD) | 62.11 (8.30) | 61.93 (9.04) | 61.56 (8.91) | 61.57 (7.26) | 64.63 (9.18) | 60.87 (6.59) | 0.203 |
| Sex |  |  |  |  |  |  | 0.033 |
| Male | 112 (46%) | 17 (35%) | 18 (37%) | 21 (43%) | 27 (56%) | 29 (60%) |  |
| Female | 131 (54%) | 32 (65%) | 31 (63%) | 28 (57%) | 21 (44%) | 19 (40%) |  |
| Education (years) | 15.62 (3.38) | 16.32 (3.28) | 15.83 (3.48) | 15.15 (3.39) | 15.39 (3.23) | 15.39 (3.56) | 0.601 |
| Smoking |  |  |  |  |  |  | >0.9 |
| Never | 92 (39%) | 19 (41%) | 17 (35%) | 20 (41%) | 17 (36%) | 19 (40%) |  |
| Smoker (current/past) | 146 (61%) | 27 (59%) | 32 (65%) | 29 (59%) | 30 (64%) | 28 (60%) |  |
| Systolic BP | 125.57 (16.86) | 121.35 (18.86) | 123.59 (16.20) | 124.47 (16.30) | 130.87 (16.18) | 127.98 (15.47) | 0.05 |
| Diastolic BP | 76.57 (8.48) | 72.49 (7.14) | 76.10 (8.30) | 77.68 (9.42) | 76.96 (7.69) | 79.87 (8.27) | <0.001 |
| Total Cholesterol | 185.64 (42.28) | 190.73 (36.02) | 189.73 (44.59) | 189.63 (43.68) | 172.94 (45.70) | 184.98 (39.66) | 0.202 |
| LDL Cholesterol | 105.59 (35.46) | 105.46 (27.83) | 107.96 (36.84) | 112.08 (37.54) | 94.98 (37.10) | 107.29 (36.11) | 0.202 |
| HDL Cholesterol | 62.58 (15.35) | 71.54 (16.53) | 64.78 (13.51) | 59.63 (13.32) | 60.00 (16.20) | 56.96 (13.06) | <0.001 |
| Triglycerides (TG) | 96.48 (56.94) | 75.06 (25.60) | 93.33 (63.96) | 100.04 (44.34) | 96.65 (51.53) | 117.29 (78.70) | 0.008 |
| Apo B | 93.56 (25.49) | 92.15 (18.35) | 93.65 (26.85) | 96.55 (27.15) | 88.02 (27.05) | 97.38 (26.76) | 0.403 |
| CRP | 2.23 (4.12) | 1.53 (1.74) | 1.35 (1.16) | 2.14 (2.17) | 2.53 (3.75) | 3.60 (7.77) | 0.054 |
| BMI | 25.68 (3.90) | 22.80 (3.32) | 24.98 (3.53) | 26.40 (3.30) | 26.40 (3.89) | 27.93 (3.60) | <0.001 |
| WC | 91.08 (11.48) | 83.63 (10.50) | 88.61 (12.37) | 92.48 (9.25) | 92.96 (11.15) | 97.64 (9.27) | <0.001 |
| Fat % | 32.86 (8.08) | 30.10 (7.78) | 34.34 (7.76) | 34.70 (8.46) | 32.81 (7.72) | 32.08 (8.13) | 0.039 |
| Previously Diagnosed CVD* | 53 (22%) | 7 (14%) | 11 (22%) | 9 (18%) | 13 (27%) | 13 (27%) | 0.503 |
| Metabolic Syndrome | 31 (13%) | 1 (2.1%) | 6 (12%) | 7 (14%) | 6 (13%) | 11 (23%) | 0.052 |

*Includes hypertension, diabetes, ischemic heart disease (IHD), or dyslipidemia.

**Appendix Table S2: Association between bias-corrected ΔAge from US, Blood and AI-ECG Biological Age clocks (per 1 SD increase) and metabolic syndrome**

| Clock | Model | N | OR per 1 SD | 95% CI | p |
| --- | --- | --- | --- | --- | --- |
| US | Unadjusted | 241 | 2.01 | 1.32–3.17 | 0.002 |
| US | Adjusted for age & sex | 241 | 2.2 | 1.41–3.58 | <0.001 |
| Blood | Unadjusted | 236 | 1.51 | 1.07–2.13 | 0.017 |
| Blood | Adjusted for age & sex | 236 | 1.53 | 1.06–2.21 | 0.022 |
| AI-ECG | Unadjusted | 177 | 1.12 | 0.72–1.73 | 0.626 |
| AI-ECG | Adjusted for age & sex | 176 | 1.19 | 0.74–1.93 | 0.472 |

**Appendix Figure S1. Bias adjustment of US-based biological age estimates.**


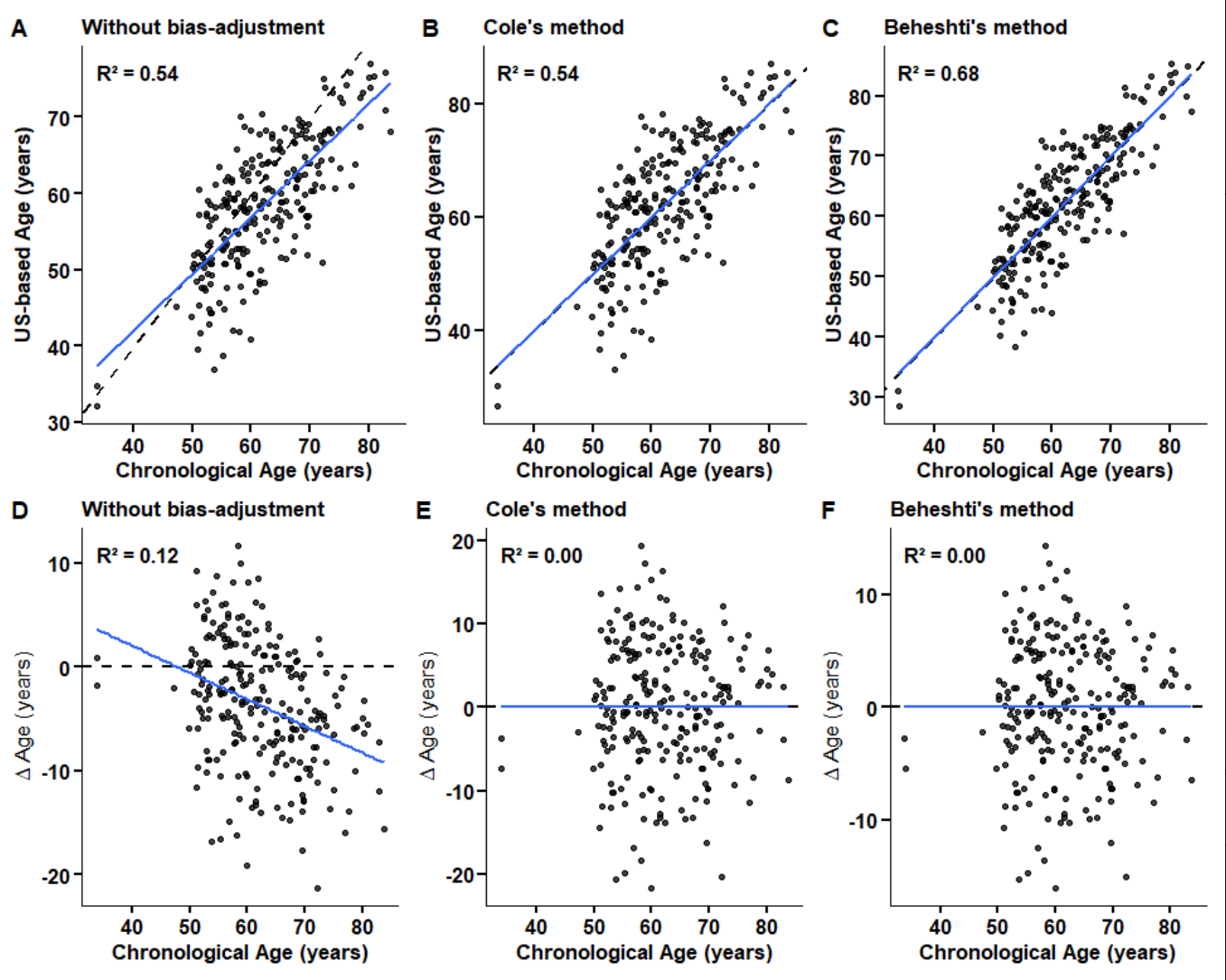


Supplementary Figure S1. Bias adjustment of US-based biological age estimates. Top row (A–C) shows scatterplots of US-based predicted age versus chronological age before bias adjustment (A), after Cole’s linear bias correction (B), and after Beheshti’s offset-based correction (C). The dashed black line represents the line of identity (y = x), and the solid blue line indicates the fitted linear regression. Bottom row (D–F) displays the corresponding age-delta (ΔAge = predicted age − chronological age) as a function of chronological age for the unadjusted model (D), Cole-corrected estimates (E), and Beheshti-corrected estimates (F). The dashed horizontal line denotes ΔAge = 0. Bias correction effectively removes the age-dependency of ΔAge, yielding age-independent residuals.
